# Pokemon inhibits Bim transcription to promote the proliferation, anti-anoikis, invasion, histological grade, and dukes stage of colorectal neoplasms

**DOI:** 10.1007/s00432-024-05904-1

**Published:** 2024-08-03

**Authors:** Yan Wang, Huiling Zeng, Li Li, Jizhen Liu, Jiantao Lin, Yanhong Bie, Sen Wang, Xiaoguang Cheng, Bayaer Nashun, Yunhong Yao, Xinrong Hu, Yi Zhao

**Affiliations:** 1 Microbiology and Immunology Department, Guangdong Medical University, Dongguan, 523808 China; 2 Pathology Department of The First Dongguan Affiliated Hospital, Guangdong Medical University, Dongguan, 523713 China; 3 Pathology Department, Huizhou Health Sciences Polytechnic, Huizhou, 516007 China; 4 Key Laboratory of Molecular Diagnosis, Guangdong Medical University, Dongguan, 523808 China; 5 Animal Center of Guangdong Medical University, Guangdong Medical University, Dongguan, 523808 China

**Keywords:** Pokemon, Bim, Colorectal neoplasms, Anoikis

## Abstract

**Purpose:**

This study aims to determine whether Pokemon regulates Bim activity in colorectal carcinoma (CRC) carcinogenesis.

**Methods:**

Clinical tissue samples were analyzed to detect the expression and clinicopathological significance of Pokemon and Bim in CRC. Proliferation, apoptosis, and invasion assays were conducted to identify the regulatory effect of Pokemon on Bim. The combined treatment effects of Pokemon knockdown and diamminedichloroplatinum (DDP) were also examined.

**Results:**

Immunohistochemical analysis of 80 samples of colorectal epithelia (CRE), 80 cases of colorectal adenoma (CRA), and 160 of CRC samples revealed protein expression rates of 23.8%, 38.8%, and 70.6% for Pokemon, and 88.8%, 73.8%, and 31.9% for Bim, respectively. A significant negative correlation was observed between Pokemon and Bim expression across the CRE, CRA, and CRC lesion stages. In CRC, higher Pokemon and lower Bim expression correlated with higher histological grades, advanced Dukes stages, and increased cancer invasion. In both LoVo and HCT116 cells, overexpression of Pokemon significantly reduced Bim expression, leading to increased proliferation, resistance to anoikis, and cell invasion. Additionally, Pokemon overexpression significantly decreased DDP-induced Bim expression, reduction of anti-apoptosis and invasion, whereas Pokemon knockdown resulted in the opposite effects.

**Conclusion:**

These findings suggest that Pokemon inhibits Bim transcription, thereby promoting CRC proliferation, resistance to apoptosis, invasion, and advancing histological grade and Dukes staging. Pokemon knockdown enhances the therapeutic efficacy of DDP in the treatment of CRC.

**Supplementary Information:**

The online version contains supplementary material available at 10.1007/s00432-024-05904-1.

## Introduction

Colorectal carcinoma (CRC) is a common malignant digestive cancer, ranking as the third most common cancer in men and the second in women worldwide (Biller and Schrag 2021; Lucchetti et al. 2017; Rossi et al. 2018). In recent years, CRC has accounted for nearly one million new cancer cases and 600,000 deaths annually, with numbers steadily increasing. The primary cause of mortality in CRC is early, undetected, and uncontrolled metastasis (Punt et al. 2017; Sa et al. 2018). Anoikis, a specialized form of programmed cell death, acts as a crucial barrier to cancer metastasis by inducing cell death when cells detach from their native extracellular matrix (Strilic and Offermanns 2017; Wang et al. 2018).

Anoikis resistance is vital for CRC cells to metastasize and establish secondary tumors. Maamer-Azzabi et al. described that the upregulation of Bcl-2 interacting mediator of cell death (Bim) expression is a critical factor in anoikis initiation in CRC cells. Bim, a member of the BH3-only subfamily of the Bcl-2 family, is a regulator of apoptosis (Shukla et al. 2017). Bim-mediated anoikis could be a pivotal mechanism in preventing CRC metastasis, yet another unidentified pathway may regulate Bim expression (Maamer-Azzabi et al. 2013).

The proto-oncogene POK erythroid myeloid ontogenic factor (Pokemon) is a member of the transcription factor BTB/POZ-ZF family (Lunardi et al. 2013; Maeda et al. 2009). Pokemon has a pleiotropic role in various biological processes and is involved in tumor occurrence and development in various types of cancer (Guo et al. 2017; Lunardi et al. 2013; Maeda et al. 2005a). Pokemon contributes to tumorigenesis by suppressing the p14ARF–MDM2–p53 pathway (Maeda et al. 2005b). Our previous research found that Pokemon overexpression in CRC tissues is related to malignant behavior, independent of the P14ARF-MDM2-P53 pathway, suggesting the possibility of another underlying mechanism (Zhao et al. 2014).

Liu et al. investigated the cancer-promoting effects of Pokemon in human hepatoma cells and non-malignant liver cell lines, demonstrating that silencing Pokemon significantly upregulates Bim expression and increases the sensitivity of hepatoma cells to anoikis, especially in the presence of chemical reagents and cell-specific culture conditions (Liu et al. 2012). Conversely, ectopic Pokemon expression in QGY7703 and HL7702 cells significantly reduced Bim expression and anoikis in non-malignant liver cells, suggesting that Pokemon suppresses Bim to prevent anoikis (Liu et al. 2012).

Our previous in vitro study showed that high Pokemon expression is associated with CRC malignancy, therefore, we hypothesized that Pokemon inhibits Bim-mediated anoikis to promote CRC growth. To the best of our knowledge, no studies have specifically examined the impact of Pokemon on Bim-mediated activity in CRC. We conducted a five-year study in patients with cancer to investigate the correlation between Pokemon and Bim protein expression. Additionally, we performed in vitro experiments to validate our clinical findings and further explore the Pokemon-Bim-Anoikis pathway. Considering the multi-pathway nature of the carcinogenic effect of Pokemon, we also assessed its effects under diamminedichloroplatinum (DDP) treatment.

This study aims to elucidate the mechanism of the Pokemon-Bim-Anoikis pathway in carcinogenesis and metastasis of CRC, particularly under challenging environments such as exposure to chemical reagents and during cell metastasis.

## Materials and methods

### Clinical specimens

Archival formalin-fixed and paraffin-embedded specimens from 160 colorectal neoplasms (CRN), 80 colorectal adenoma (CRA), and 80 colorectal epithelium (CRE) samples were collected from the Affiliated Hospital of Guangdong Medical University (Zhanjiang, China) between 2009 and 2015. The median age of the patients was 60 years (range: 26–89). All samples were obtained before clinical treatments (chemotherapy or radiotherapy). The study protocol for human experiments was approved by the ethics committee of the hospital. This study adhered to the standards set by the Declaration of Helsinki. All patients were informed and consented to the use of their specimens for clinical diagnosis, treatment, and scientific research. The diagnosis of CRN was confirmed by two senior pathologists according to the latest World Health Organization (WHO) criteria and American Clinical Practice Guidelines (Benson et al. 2017; Vogel et al. 2017).

### Immunohistochemistry

Immunohistochemistry (IHC) was performed following previously described protocols (Guo et al. 2017; Wu et al. 2013; Zhao et al. 2014). The streptavidin-peroxidase immunohistochemical staining kit (SP-0023) was obtained from Biosynthesis Biotechnology Co., Ltd. (Beijing, China). Anti-Pokemon polyclonal antibody (ab70208, 1:400; Abcam, Cambridge, UK) and anti-Bim monoclonal antibody (#2933, 1:400; Cell Signaling Technology, Danvers, MA, USA) were used for IHC. Phosphate-buffered saline (PBS) was used instead of the primary antibodies as the blank control. The staining results were evaluated and scored independently by two expert pathologists under double-blinded conditions, as described by Shimizu et al. (Shimizu et al. 1990; Zhao et al. 2014).

### Cell lines and cell culture

Two human CRN cell lines, LoVo (ATCC^®^CCL-229) and HCT116 (ATCC^®^CCL-247), were used for in vitro studies. LoVo-PKD cells (stable knockdown of the Pokemon gene in LoVo cells) and LoVo-NC cells (control cells in the Pokemon knockdown assay) were obtained from the Institute of Oncology, Guangdong Medical University (Zhao et al. 2014). HCT116-P cells (HCT116 cells stably expressing Pokemon) and HCT116-NC cells (control cells transfected with empty vectors) were obtained from Shanghai Gene Chem Co. Ltd (Shanghai, China). The cells were cultured in standard growth medium.

### Plasmid construction

The GV-144-Pokemon plasmid was constructed by inserting a full-length cDNA fragment, retro-transcribed from Pokemon mRNA (GenBank accession number: NM_015898) (Zhao et al. 2014; Zhu et al. 2017), into the GV-144 commercial transformation plasmid (Shanghai Gene Chem Co. Ltd). The plasmid included coding sequences for EGFP, Kana, and Neo et al., which were used for screening and tracking transfection efficiency in both prokaryotic and eukaryotic cells.

### Pokemon stable expression cell lines

Before transfection, HCT116 cells were seeded onto six-well plates at a density of 1 × 10^5^ cells per well in serum-free medium without antibiotics and cultured overnight. Lipofectamine 3000 (10 µL; Thermo Fisher Scientific, Guangzhou, Guangdong, China) was used to transfect 4 µg of each plasmid per well (GV-144-Pokemon or GV-144). Transfected cells were incubated with G418 (500 ng/mL) for 14 days to establish a stable Pokemon expression cell line (HCT116-P cells) and an empty control cell line (HCT116-NC). Pokemon expression was validated using qRT-PCR and western blotting.

### RNA extraction and qRT-PCR

Total RNA was extracted from cells using a TRIzol reagent kit (Thermo Fisher Scientific, Waltham, MA, USA), following manufacturer’s instructions. cDNA was synthesized from 1 µg of total RNA using the M-MLV First-strand System for qRT-PCR Kit (Thermo Fisher Scientific) following manufacturer’s instructions. qRT-PCR was performed using FastStart Universal SYBR Green Master Mix (Roche, Shanghai, China) and repeated three times.

The primer sequences used for qRT-PCR are listed in Table S1. PCR cycling conditions were: pre-denaturation at 50℃ for 2 min, denaturation at 95℃ for 10 min, followed by 40 cycles of denaturation at 95℃ for 15 s and annealing at 60℃ for the 60 s.

### Western blot assay

Total protein was extracted using the Radio-Immunoprecipitation Assay (RIPA) Lysis Buffer system (sc24948, Santa Cruz, Shanghai, China). Protein concentrations were determined using a BCA Protein Quantification Kit (ab102536; Abcam, Cambridge, UK), following manufacturer’s instructions. Proteins (20 µg) were separated on a 10% SDS/PAGE under denatured reducing conditions and transferred to PVDF membranes. Membranes were blocked with 5% non-fat dried milk for 2 h and incubated overnight at 4℃ with primary antibodies. After washing with cold TBST, membranes were incubated with secondary antibodies (ab191866; Abcam, Cambridge, UK) for 1 h. Signals were detected using a western blot luminol reagent (sc2048, Santa Cruz, Shanghai, China). Primary antibodies used were anti-Pokemon (ab70208, Abcam, Cambridge, UK), anti-Bim (#2933, cell signal technology, Guangzhou, China), and anti-(β-actin) (sc8432, Santa Cruz, Shanghai, China). This experiment was repeated thrice.

### Cell proliferation assay

A Cell Counting Kit 8 (CCK8; Sigma, St. Louis, MO, USA) was used to measure cell proliferation. Absorbance at 450 nm was measured using a BioTek Synergy 2 automatic enzyme-labeled meter (Biotek, USA). Each cell proliferation assay was repeated thrice.

### Transwell migration assay

Cell migration was assessed using Transwell permeable support system (6.5 mm in diameter, 8 μm pore size, Corning Costar Corp., Shenzhen, China). Following manufacturer’s instructions, cells were suspended in serum-free Dulbecco’s modified Eagle’s medium (DMEM), and 0.2 mL of the cell suspension (1 × 10^4^ cells/well) was added to the upper chamber of the Transwell system. The lower chamber contained 0.6 mL of DMEM supplemented with 10% fetal bovine serum as chemoattractant. After 48 h of culture, non-migrating cells were removed from the upper surface of the membrane using cotton swabs. Cells that had migrated to the bottom of the membrane were stained with 0.1% crystal violet solution for 30 min at 37℃, rinsed twice with PBS, and counted under a microscope (×100) in four randomly selected visual fields. Results were expressed as means ± SD, with each experiment repeated three times.

### Anoikis and apoptosis assays

After 48 h in culture, cells were harvested, washed twice with cold PBS, and fixed in 70% alcohol at -20℃ overnight. Apoptotic rates were determined using the Annexin V-FITC apoptosis kit (E606336, Sangon Biotech Co., Ltd., Shanghai, China) and analyzed with the FACSCanto II system (BD, CA, USA).

For the anoikis assay, cells were seeded into six-well plates (Corning, NY, USA) with a low attachment surface area (to prevent cell aggregation) in standard growth media (Chen et al. 2018). Non-adherent cells were collected for flow cytometry, while adherent cells in CellBIND^®^ surface plates served as controls. Experiments were performed in according to the manufacturer’s instructions and repeated three times.

### Treatment of cells with chemotherapeutic agents

LoVo cells were divided into three groups: LoVo-NC, LoVo-NC + DDP, and LoVo-PKD + DDP. HCT116 cells were divided into three groups: HCT116-NC, HCT116-NC + DDP, and HCT116-P + DDP. The LoVo-NC + DDP, LoVo-PKD + DDP, HCT116-NC + DDP, and HCT116-P + DDP groups were treated with DDP (1.0 µg/mL) for 24 h, after which the expression of Bim, cell apoptosis, and invasion were assessed.

### Statistical analysis

Data are expressed as the means ± SD of three independent experiments. Statistical analyses were performed using SPSS 17.0, including one-way analysis of variance (ANOVA). Measurement data were compared using the LSD and SNK methods of analysis of variance. Correlation and chi-square tests were also applied. A p-value of < 0.05 was considered statistically significant, and p-value of < 0.01 was considered highly significant.

## Results

### Protein expression of Pokemon and Bim in CRE, CRA, and CRN

**Fig. 1:**
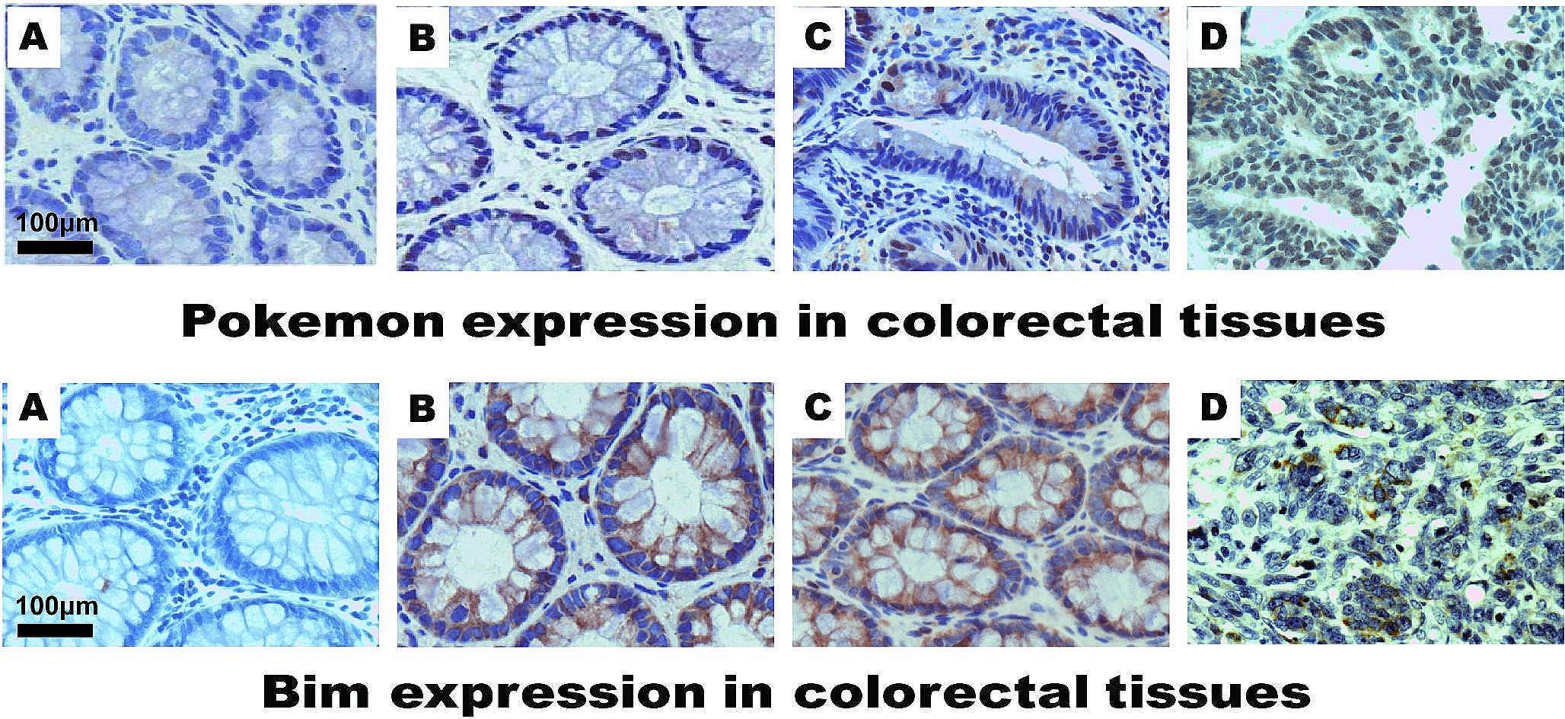
Immunohistochemistry staining of Pokemon and Bim in CRE, CRA, and CRC samples (SP × 400). **A**: Blank control; **B**: Positive staining in CRE; **C**: Positive staining in CRA; **D**: Positive staining in CRC

Immunohistochemical analysis showed positive staining for Pokemon and Bim in the nuclei and/or cytoplasm of cells (Fig. 1), whereas cells in the blank control group (no primary antibodies) exhibited no staining. The expression rates of Pokemon in CRE, CRA, and CRN were 23.8%, 38.8%, and 70.6% (*P* < 0.05), respectively. Conversely, the expression rates of Bim were 88.8%, 73.8%, and 31.9% (*P* < 0.05), respectively (Table 1). The results showed that the high expression rate of Pokemon and the low expression rate of Bim were observed in CRN tissues compared with CRE and CRA tissues.

**Table 1:** The expression rates (%) of Pokemon and Bim proteins in CRE, CRA and CRC tissues

Tissues	Case	Pokemon	Bim
	no.	% (P/T)	% (P/T)
CRE	80	23.8 (19/80)	88.8 (71/80)
CRA	80	38.8 (31/80)*	73.8 (59/80)*
CRC	160	70.6 (113/160)^‡,#^	31.9 (51/160)^‡,#^

P, positive case number; T, total case number

*, *P* < 0.05 between CRE and CRA; ‡,*P* < 0.05 between CRE and CRC; #, *P* < 0.05 between CRA and CRC

### Correlation between the expression of Pokemon and Bim and the clinicopathological characteristics of CRN

The expression of Pokemon was significantly higher in colon cancer than that in rectal cancer, in high-grade cancer than that in low-grade cancer, in deep invasive cancer than that in shallow invasive cancer, and in cancers with later Dukes stages than that in cancers with earlier Dukes stages (Table 2). Conversely, the expression of Bim was significantly lower in colon cancer than that in rectal cancer, in high-grade cancer than that in low-grade cancer, in late Dukes stage cancer than that in early Dukes stage cancer, and in deep invasive cancer than that in shallow invasive cancer. There was no significant association between the expression of Pokemon and Bim and the sex or age of patients.

**Table 2:** The relationship between the expression of Pokemon, Bim and the clinicopathological characteristics in CRC

Clinicopathological characteristics	Informative cases	Pokemon			*P* value	Bim			*P* value
		Positive cases	Positive rate(%)			Positive cases		Positive rate(%)	
**Sex**									
M	89	68		76.4	0.072	29	32.6		0.829
F	71	45		63.4		22	31.0		
**Age**									
≤ 60	85	61		71.8	0.736	31	36.5		0.184
>60	75	52		69.3		20	26.7		
**Tumor location**									
colon	88	68		77.3	0.041	22	25.0		0.039
rectum	72	45		62.5		29	40.3		
**Histological grade**									
G1	35	17		48.6	0.012	13	37.1		0.020
G2	85	64		75.3		33	38.8		
G3	23	19		82.6		4	17.4		
mucinous adenocarcinoma	17	13		76.5		1	5.9		
**Duke’s stage**									
A + B	78	49		62.8	0.035	31	39.7		0.037
C + D	82	64		78.0		20	24.4		
**Invasive depth**									
muscularis	23	12		52.2	0.036	12	52.2		0.024
serosa	137	101		73.7		39	28.5		

### Relationship between the expression of Pokemon and Bim in CRN

A negative correlation was observed between Pokemon and Bim expression in CRN tissues, with a correlation coefficient of -0.203 (*P* < 0.05) (Table S2).

### Pokemon inhibited Bim mRNA and protein levels in CRC cells

**Fig. 2:**
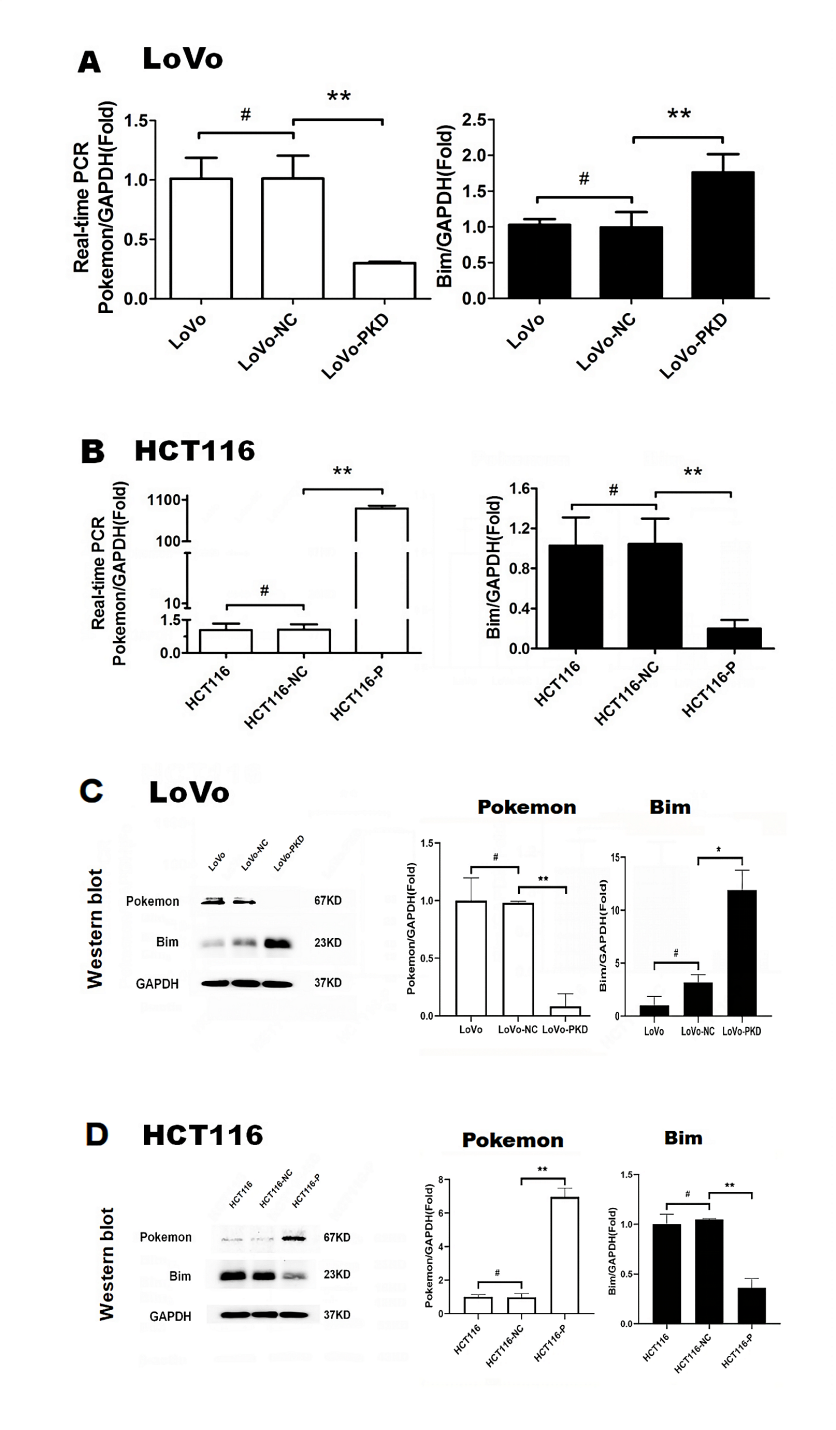
Pokemon downregulates Bim mRNA and protein levels in CRC cells. **A**: Knockdown of Pokemon upregulates mRNA expression of Bim. Groups: LoVo (untreated), LoVo-NC (negative control), and LoVo-PKD (Pokemon knockdown). **B**: Pokemon overexpression inhibits Bim mRNA expression. Groups: HCT116 (untreated), HCT116-NC (negative control), and HCT116-P (Pokemon overexpression). **C**: Stable knockdown of Pokemon increases Bim protein expression. **D**: Stable overexpression of Pokemon downregulates Bim protein expression. ^#^*P* > 0.05; *P < 0.05; **P < 0.01

LoVo cells in the LoVo (untreated control), LoVo-NC (negative control), and LoVo-PKD (stable knockdown of Pokemon) groups were cultured for 48 h. To further investigate the effect of Pokemon on Bim expression, we ectopically overexpressed Pokemon in HCT116 cells, which normally have low endogenous expression of Pokemon. HCT116 cells in the HCT116 (untreated control), HCT116-NC (negative control), and HCT116-p groups (stably overexpressing Pokemon) were cultured for 48 h. qRT-results (Fig. 2A) showed similar mRNA levels of Pokemon and Bim in the LoVo-NC and LoVo groups. In contrast, the mRNA levels of Pokemon in the LoVo-PKD group decreased by 70.0%, while Bim increased by 76.2% compared to those in the LoVo-NC group. Figure 2B showed no detectable Pokemon mRNA in HCT116 and HCT116-NC and no differences in Bim mRNA expression between these two groups. Moreover, mRNA levels of Pokemon were significantly increased in the HCT116-p group, while mRNA levels of Bim decreased by 79.8% in this group compared to the HCT116-NC group.

Western blotting (Fig. 2C) revealed similar Pokemon protein levels in the LoVo-NC and LoVo groups, with a reduction of 90.0% in the LoVo-PKD group compared to the LoVo-NC group, whereas Bim protein levels increased 8.76-fold. Figure 2D showed minimal protein levels of Pokemon in the HCT116 and HCT116-NC groups, and similar Bim protein expression between the two groups (Fig. 2D). The protein levels of Pokemon in the HCT116-p group significantly increased, while Bim protein levels were decreased by 68.85% compared to those in the HCT116-NC group.

### Pokemon promoted cell proliferation, reduced apoptosis, and increased invasion in CRC cells

**Fig. 3:**
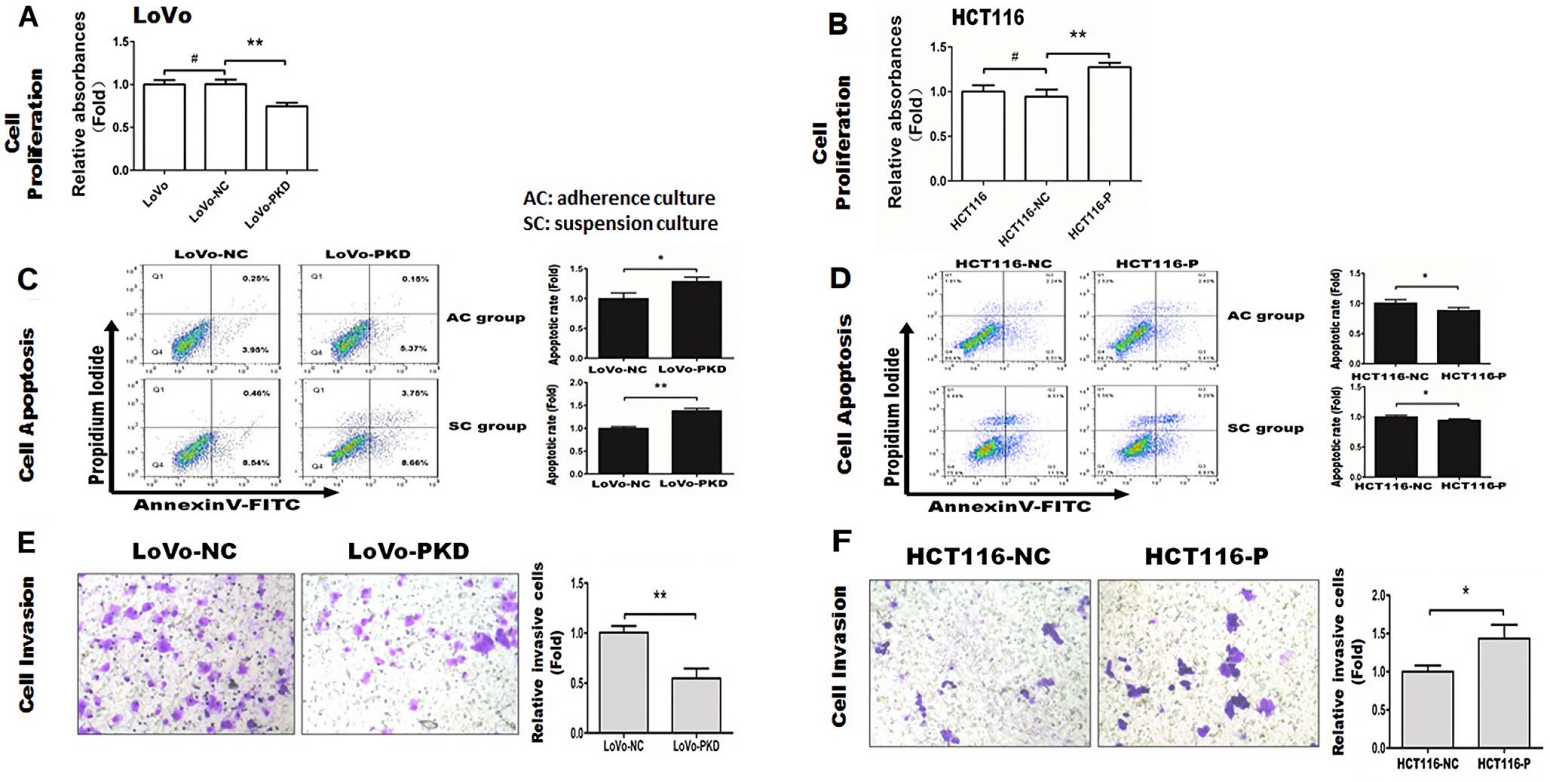
Pokemon promoted cell proliferation, reduced apoptosis, and increased invasion in CRC cells. **A**, **B**: CCK8 assays in LoVo and HCT116 cells with Pokemon knockdown or overexpression, respectively. **A**: LoVo (untreated control), LoVo-NC (negative control) and LoVo-PKD (Pokemon knockdown) ^#^*P* > 0.05. **P < 0.01. **B**: HCT116 (untreated control), HCT116-NC (negative control), and HCT116-P (Pokemon overexpression). ^#^*P* > 0.05. **P < 0.01. **C**, **D**: Cell apoptosis was evaluated using flow cytometer. AC: adherent culture; SC: suspension culture. **C**: *P < 0.05, **P < 0.01 vs. LoVo-NC group. **D**: *P < 0.05, **P < 0.01 vs. HCT116-NC cell. **E**, **F**: Effects of Pokemon on invasion in CRC cells using invasion assays. *P < 0.05; **P < 0.01

Cell proliferation assays (Fig. 3A) showed similar proliferation rates in the LoVo-NC and LoVo groups (*P* > 0.05). Conversely, proliferation in the LoVo-PKD group significantly decreased by 36.6% (*P* < 0.01) at 48 h compared with that in the LoVo-NC group. Moreover, cell proliferation rates were similar in both HCT116-NC and HCT116 groups (*P* > 0.05, Fig. 3B), whereas the proliferation rate in the HCT116-P group increased by 41.9% (*P* < 0.01) at 48 h compared with that in the HCT116-NC group.

Apoptosis assays (Fig. 3C) demonstrated that the apoptosis rate in the LoVo-PKD group increased by 1.28-fold under adherent culture conditions (*P* < 0.05) and by 1.38-fold under low-adhesion conditions (*P* < 0.01) compared to the LoVo-NC group. In addition, as presented in Fig. 3D, a 0.22-fold decrease(*P* < 0.05) in the apoptosis rate of the HCT116-P group under adherent culture conditions, compared to that in the HCT116-NC group. Transitional growth in SC significantly promoted cell anoikis. However, the number of cells undergoing anoikis decreased by 1.68% in the HCT116-P group (*P* < 0.05) compared to that in the HCT116-NC group.

Transwell migration assays (Fig. 3E) demonstrated that cell invasion in the LoVo-PKD decreased by 45.4% (*P* < 0.01) compared to that in the LoVo-NC group. Figure 3F presented an increase of 43.6% (*P* < 0.05) in cell invasion of HCT116-P cells compared to that in the HCT116-NC group.

### Pokemon knockdown enhances DDP-induced Bim expression, anoikis, and suppression of invasion, whereas Pokemon Overexpression has opposite effects

**Fig. 4:**
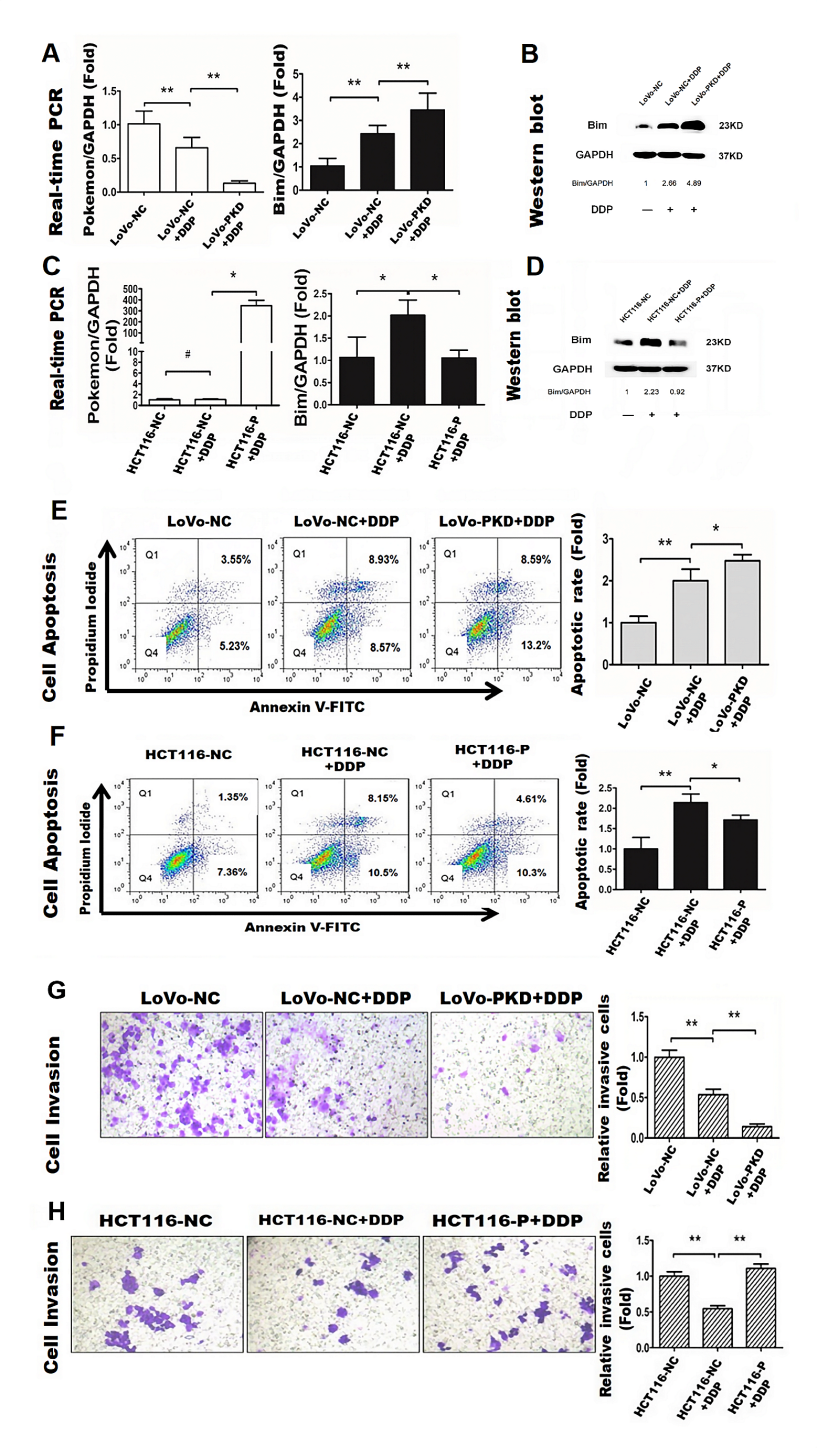
Pokemon knockdown enhances DDP-induced effects on CRC cells. **A**, **B**: Pokemon knockdown increases DDP-induced Bim expression. Groups: LoVo-NC (negative control), LoVo-NC + DDP (treated with DDP, 1.0 µg/mL), and LoVo-PKD + DDP (Pokemon knockdown plus DDP treatment). *P < 0.05. **C**, **D**: Pokemon overexpression counteracts DDP-induced Bim expression. Groups: HCT116-NC (negative control), HCT116-NC + DDP (treated with DDP, 1.0 µg/mL), and HCT116-P + DDP (Pokemon overexpression plus DDP treatment). *P < 0.05. **E**: Pokemon knockdown increases DDP-induced anoikis. *P < 0.05, **P < 0.01. **F**: Pokemon overexpression inhibits the DDP-induced promotion of anoikis. **P < 0.01. *P < 0.05. **G**: Pokemon knockdown augments DDP-induced downregulation of invasive ability. **P < 0.01. **H**: Pokemon overexpression antagonizes the decrease of invasive activity induced by DDP in HCT116 cells. **P < 0.01

In LoVo cells treated with DDP (1.0 µg/mL) (Fig. 4A, B), qRT-PCR results showed that Pokemon mRNA levels decreased by 35.5% and 88.2% in the LoVo-NC + DDP and LoVo-PKD + DDP groups, respectively. Bim mRNA and protein levels in the LoVo-NC + DDP group increased by 138.2% (Fig. 4A) and 166.0% (Fig. 4B), respectively, and in the LoVo-PKD group by 98.7% (Fig. 4A) and 223.0% (Fig. 4B), respectively (*P* < 0.01). Moreover, In HCT116-NC or HCT116-NC + DDP cells, Pokemon mRNA was not detected (Fig. 4C). HCT116 cells treated with DDP (1.0 µg/mL) exhibited increased Bim mRNA (95.0%) and protein expression (123.0%) (Fig. 4C, D), respectively. However, Bim mRNA and protein levels decreased by 48.1% (Fig. 4C) and 131.0% (Fig. 4D), respectively, in the HCT116-P + DDP group compared to those in the HCT116-NC + DDP group.

Subsequently, Anoikis assay displayed that anoikis rates (Fig. 4E) increased by 1-fold and 1.48-fold (*P* < 0.01) in the LoVo-NC + DDP and LoVo-PKD + DDP groups, respectively, compared to that in the LoVo-NC group. Conversely, Anoikis rate increased by 1.14-fold (*P* < 0.01) in the HCT116-NC + DDP group (Fig. 4F), but decreased by 20.0% (*P* < 0.05) in the HCT116-P + DDP group compared to the HCT116-NC + DDP group.

Additionally, Invasive activity decreased by 46.3% and 85.9% in the LoVo-NC + DDP and LoVo-PKD + DDP groups (Fig. 4G) respectively, compared to that in the LoVo-NC group. Invasive activity in the HCT116-NC + DDP group was reduced by 45.5% (*P* < 0.01) (Fig. 4H), but increased by 10.8% in the HCT116-P + DDP group (*P* > 0.05) compared to that the HCT116-NC group.

## Discussion

Pokemon protein, known for its diverse roles in various human carcinomas, including non-small cell lung cancer, laryngeal squamous cell carcinoma, and liver cancer (Gao et al. 2013; Koken et al. 1997; Liu et al. 2013; Zhao et al. 2008, 2014), has been implicated with the development and progression of CRC (Wang et al. 2010; Zhao et al. 2013). Our results (Table 2; Fig. 1) revealed a significance association between the expression of Pokemon and increasing malignancy in CRC, especially in differentiation and histological grade. The higher the expression of Pokemon, the lower the degree of tissue differentiation. This aligns with previous findings in breast adenocarcinoma (Aggarwal et al. 2010; Zu et al. 2011), suggesting that Pokemon is associated with CRC progression through the regulation of cell differentiation.

Moreover, we observed that Pokemon expression positively correlates with invasion depth and Dukes staging, suggesting its involvement in tumor metastasis and invasion. Additionally, Bim expression exhibited a negative correlation with tumor differentiation, showing higher expression in differentiated tumor tissues. Bim expression was therefore inversely proportional to tissue malignancy and was specific to tumor location (colon or rectum). These findings are consistent with studies highlighting the clinical and epidemiological characteristics associated with a decline in rectal cancer rate and an increase in right-sided colon cancer (Xu et al. 2006). Bim expression also negatively correlated with histological grade and with Pokemon expression. Furthermore, we observed a significant negative correlation between Pokemon and Bim expression and the invasion depth and Dukes stages.

There POK family members, characterized by a BTB domain and Kruppel zinc finger structure, are pivotal in embryonic development, cell differentiation, tumor formation, and metastasis (Lunardi et al. 2013; Maeda et al. 2005a). In several cellular contexts, including mouse embryonic fibroblasts, fetal liver cells, and bone marrow cells, Pokemon has been shown to bind directly to the Bim promoter, repressing Bim transcription, and thereby inhibiting Bim-mediated apoptosis (and possibly anoikis) (Liu et al. 2012; Maeda et al. 2005b, 2009). However, the specific impact of Pokemon-Bim interaction on anoikis and metastasis in CRC remains unclear. Based on the results of our histological experiments, we hypothesize that Pokemon promotes malignancy and metastasis of CRC by inhibiting Bim expression and therefore inducing antagonistic anoikis.

We testes this hypothesis in LoVo and HCT116 cells, representing metastatic CRC and primary rectal cancer cells respectively. While LoVo cells expressed Pokemon, HCT116 cells did not show expression of Pokemon. Moreover, Pokemon knockdown in LoVo cells and overexpression in HCT116 cells demonstrated a negative correlation between Pokemon and Bim mRNA expression levels.

Additionally, we observed that Pokemon expression was positively correlated with proliferation and invasion, and negatively correlated with apoptosis rate in colon cancer cells. These findings underscore the functional relevance of Pokemon and Bim in influencing CRC cell behavior, reinforcing our clinical and histological observations.

Anoikis is a form of apoptosis triggered by loss of cell adhesion to the extracellular matrix or neighboring cells (Nagaprashantha et al. 2011; Oudenaarden et al. 2018). Anoikis plays a critical regulatory role in lumen formation in glandular epithelial structures and has been implicated in tumorigenesis, metastasis, and invasive tumor formation (Oudenaarden et al. 2018; Whelan et al. 2010). Wendt et al. used caspase-3/7, evaluated focal adhesion kinase (FAK), p130Cas cleavage, DNA fragmentation, and conducted cell survival assays to study anoikis (Wendt et al. 2008). Sinicrope et al. examined tumor tissues of patients with stage II and III CRC found that elevated Bim expression correlated significantly with improved disease-free survival and overall survival (OS) compared to patients with low Bim expression. This association was particularly strong in patients with curatively resected stage II and III colon cancers treated with 5-FU-based adjuvant therapy. Multivariate Cox analysis identified Bim expression as an independent predictor of OS after adjusting for histological grade, tumor stage, age, and treatment. Their findings underscored Bim-mediated anoikis as a mechanism involved in tumor metastasis, thereby validating Bim as a tumor suppressor (Sinicrope et al. 2008). These results were consistent with observations in a mouse xenograft model (Tan et al. 2005),where Bim-deficient (BIM−/−) mice exhibited enhanced epithelial tumor growth and reduced paclitaxel-induced apoptosis compared to wild-type Bim (BIM+/+) mice (Tan et al. 2005). As demonstrated in previous studies, Pokemon promotes CRC by suppressing Bim-mediated anoikis.

We further investigated the influence of DDP, a commonly used chemotherapeutic agent, on Pokemon-mediated regulation of Bim protein levels. Stress stimuli, such as ultraviolet irradiation or chemotherapeutic effects, can activate cellular signaling pathways, such as the JNK pathway, leading to increased phosphorylation and downstream expression of Bim (Li et al. 2010; Liu et al. 2012; Song et al. 2018). Bim regulation at translational level involves two potential mechanisms: inhibition of protein synthesis or increased protein degradation. The balance of Bim proten accumulation and degradation is closely linked to apoptosis and influenced by Pokemon expression in hepatoma carcinoma cells (Liu et al. 2012). Pokemon knockdown effectively increased Bim expression in LoVo following DDP treatment, suggesting that DDP can modulate Bim protein levels through Pokemon regulation. DDP also modifies cell anoikis and migration, these findings align with clinical histology results and experimental models, underscoring the potential therapeutic implications of targeting Pokemon-Bim axis in CRC.

To the best of our knowledge, few clinical studies have and explored the clinicopathological significance of Bim in CRC, especially in relation to pathological staging and metastasis. Integration of clinical data with experimental insights into Pokemon-Bim interactions might elucidate the molecular mechanism underlying CRC metastasis, especially under stress conditions such as chemotherapy. In conclusion, our study establishes the negative correlation between Pokemon and Bim expression as pivotal for occurrence and development of CRC. Pokemon regulates Bim transcription and CRC malignancy through the inhibition of anoikis. Moreover, we found that DDP intervention can regulate Bim protein levels via Pokemon, offering novel therapeutic avenues for clinical application, particularly in knocking down Pokemon combined with DDP.

## Electronic supplementary material

Below is the link to the electronic supplementary material.

Supplementary Material 1

Supplementary Material 2

Supplementary Material 3: Table S1. The sequences of primers used for qRT-PCR. Table S2. The pearson correlation analysis of expression between Pokemon and Bim in CRN

## Data Availability

No datasets were generated or analysed during the current study.

## References

[CR1] Aggarwal A et al (2010) Expression of leukemia/lymphoma-related factor (LRF/POKEMON) in human breast carcinoma and other cancers. Exp Mol Pathol 89:140–148 20471975 10.1016/j.yexmp.2010.05.002 PMC2939325

[CR2] Benson AB 3rd, et al (2017) Colon cancer, Version 1.2017, NCCN Clinical Practice guidelines in Oncology. J Natl Compr Canc Netw 15:370–398 28275037 10.6004/jnccn.2017.0036

[CR3] Biller LH, Schrag D (2021) Diagnosis and treatment of metastatic colorectal Cancer: a review. JAMA 325:669–685 33591350 10.1001/jama.2021.0106

[CR4] Chen F et al (2018) HCRP-1 regulates EGFR-AKT-BIM-mediated anoikis resistance and serves as a prognostic marker in human colon cancer. Cell Death Dis 9:1176 30518879 10.1038/s41419-018-1217-2 PMC6281589

[CR5] Gao ZY et al (2013) The expression and clinical significance of proto-oncogene Pokemon in peripheral blood of hepatocellular carcinoma patients. Chin J Integr Traditional Western Med Liver Dis 23:239–240

[CR6] Guo C et al (2017) The expression and clinical significance of ZBTB7 in transitional cell carcinoma of the bladder. Oncol Lett 14:4857–4862 29085492 10.3892/ol.2017.6814 PMC5649710

[CR7] Koken MH et al (1997) Leukemia-associated retinoic acid receptor alpha fusion partners, PML and PLZF, heterodimerize and colocalize to nuclear bodies. Proc Natl Acad Sci U S A 94:10255–10260 9294197 10.1073/pnas.94.19.10255 PMC23349

[CR8] Li H et al (2010) Inhibition of the JNK/Bim pathway by Hsp70 prevents Bax activation in UV-induced apoptosis. FEBS Lett 584:4672–4678 21034742 10.1016/j.febslet.2010.10.050 PMC3397246

[CR9] Liu K et al (2012) Pokemon silencing leads to Bim-mediated anoikis of human hepatoma cell QGY7703. Int J Mol Sci 13:5818–5831 22754333 10.3390/ijms13055818 PMC3382817

[CR10] Liu LY et al (2013) The expression of Pokemon and E2F3 in laryngeal squamous cell carcinoma and their clinical significance. J Audiol Speech Pathol 21:258–262

[CR11] Lucchetti D et al (2017) Differentiation affects the release of exosomes from Colon cancer cells and their ability to modulate the behavior of recipient cells. Am J Pathol 187:1633–1647 28619275 10.1016/j.ajpath.2017.03.015

[CR12] Lunardi A et al (2013) Role of LRF/Pokemon in lineage fate decisions. Blood 121:2845–2853 23396304 10.1182/blood-2012-11-292037 PMC3624932

[CR13] Maamer-Azzabi A et al (2013) Metastatic SW620 colon cancer cells are primed for death when detached and can be sensitized to anoikis by the BH3-mimetic ABT-737. Cell Death Dis 4, e801 10.1038/cddis.2013.328 PMC3789186 24030153

[CR14] Maeda T et al (2005a) Role of the proto-oncogene Pokemon in cellular transformation and ARF repression. Nature 433:278–285 15662416 10.1038/nature03203

[CR15] Maeda T et al (2005b) The transcription factor Pokemon: a new key player in cancer pathogenesis. Cancer Res 65:8575–8578 16204018 10.1158/0008-5472.CAN-05-1055

[CR16] Maeda T et al (2009) LRF is an essential downstream target of GATA1 in erythroid development and regulates BIM-dependent apoptosis. Dev Cell 17:527–540 19853566 10.1016/j.devcel.2009.09.005 PMC3134301

[CR17] Nagaprashantha LD et al (2011) The sensors and regulators of cell-matrix surveillance in anoikis resistance of tumors. Int J Cancer 128:743–752 20949625 10.1002/ijc.25725 PMC3292620

[CR18] Oudenaarden CRL et al (2018) Re-inforcing the cell death army in the fight against breast cancer. J Cell Sci 131 10.1242/jcs.212563 30139926

[CR19] Punt CJ et al (2017) From tumour heterogeneity to advances in precision treatment of colorectal cancer. Nat Rev Clin Oncol 14:235–246 27922044 10.1038/nrclinonc.2016.171

[CR20] Rossi M et al (2018) Colorectal Cancer and Alcohol Consumption-Populations to Molecules. Cancers (Basel). 10 10.3390/cancers10020038 PMC5836070 29385712

[CR21] Sa KD et al (2018) A miR-124/ITGA3 axis contributes to colorectal cancer metastasis by regulating anoikis susceptibility. Biochem Biophys Res Commun 501:758–764 29758195 10.1016/j.bbrc.2018.05.062

[CR22] Shimizu M et al (1990) Immunohistochemical staining of Ha-ras oncogene product in normal, benign, and malignant human pancreatic tissues. Hum Pathol 21:607–612 2161789 10.1016/s0046-8177(96)90006-4

[CR23] Shukla S et al (2017) BH3-only protein BIM: an emerging target in chemotherapy. Eur J Cell Biol 96:728–738 29100606 10.1016/j.ejcb.2017.09.002

[CR24] Sinicrope FA et al (2008) Prognostic impact of bim, puma, and noxa expression in human colon carcinomas. Clin Cancer Res 14:5810–5818 18794091 10.1158/1078-0432.CCR-07-5202 PMC2948480

[CR25] Song T et al (2018) Identification of JNK1 as a predicting biomarker for ABT-199 and paclitaxel combination treatment. Biochem Pharmacol 155:102–109 29953843 10.1016/j.bcp.2018.06.019

[CR26] Strilic B, Offermanns S (2017) Intravascular survival and extravasation of Tumor cells. Cancer Cell 32:282–293 28898694 10.1016/j.ccell.2017.07.001

[CR27] Tan TT et al (2005) Key roles of BIM-driven apoptosis in epithelial tumors and rational chemotherapy. Cancer Cell 7:227–238 15766661 10.1016/j.ccr.2005.02.008

[CR28] Vogel JD et al (2017) The American Society of Colon and rectal surgeons Clinical Practice guidelines for the treatment of Colon cancer. Dis Colon Rectum 60:999–1017 28891842 10.1097/DCR.0000000000000926

[CR29] Wang B et al (2010) Expression of Pokemon and its clinical significance in human hepatocellular carcinoma. Chin J Gastroenterol Hepatol 19:151–153

[CR30] Wang YN et al (2018) CPT1A-mediated fatty acid oxidation promotes colorectal cancer cell metastasis by inhibiting anoikis. Oncogene 37:6025–6040 29995871 10.1038/s41388-018-0384-z

[CR31] Wendt MK et al (2008) Constitutive CXCL12 expression induces anoikis in colorectal carcinoma cells. Gastroenterology 135:508–517 18558091 10.1053/j.gastro.2008.05.033 PMC2583344

[CR32] Whelan KA et al (2010) Hypoxia suppression of Bim and Bmf blocks anoikis and luminal clearing during mammary morphogenesis. Mol Biol Cell 21:3829–3837 20861305 10.1091/mbc.E10-04-0353 PMC2982135

[CR33] Wu M et al (2013) EIF4E over-expresses and enhances cell proliferation and cell cycle progression in nasopharyngeal carcinoma. Med Oncol 30:400 23277284 10.1007/s12032-012-0400-z

[CR34] Xu A et al (2006) The trend of clinical characteristics of colorectal cancer during the past 20 years in Guangdong Province. Natl Med J China 86:272–275 16677509

[CR37] Zhao ZH et al (2008) Overexpression of Pokemon in non-small cell lung cancer and foreshowing tumor biological behavior as well as clinical results. Lung Cancer 62:113–119 18550205 10.1016/j.lungcan.2008.02.014

[CR35] Zhao GT et al (2013) Expression of the proto-oncogene Pokemon in Colorectal Cancer - Inhibitory effects of an siRNA. Asian Pac J Cancer Prev 14:4999–5005 24175766 10.7314/apjcp.2013.14.9.4999

[CR36] Zhao Y et al (2014) Pokemon enhances proliferation, cell cycle progression and anti-apoptosis activity of colorectal cancer independently of p14ARF-MDM2-p53 pathway. Med Oncol 31:288 25367850 10.1007/s12032-014-0288-x

[CR38] Zhu M et al (2017) LRF inhibits p53 expression in colon cancer cells via modulating DAP5 activity. Cell Biochem Funct 35:401–406 28849590 10.1002/cbf.3287

[CR39] Zu X et al (2011) Pro-oncogene Pokemon promotes breast cancer progression by upregulating survivin expression. Breast Cancer Res 13:R26 21392388 10.1186/bcr2843 PMC3219187

