# Supplementary figures and images for: Pokemon inhibits Bim transcription to promote the proliferation, anti-anoikis, invasion, histological grade, and dukes stage of colorectal neoplasms
Source: J Cancer Res Clin Oncol. 2024 Aug 3;150(8):380. doi: 10.1007/s00432-024-05904-1 (PMC11297103; doi:10.1007/s00432-024-05904-1)

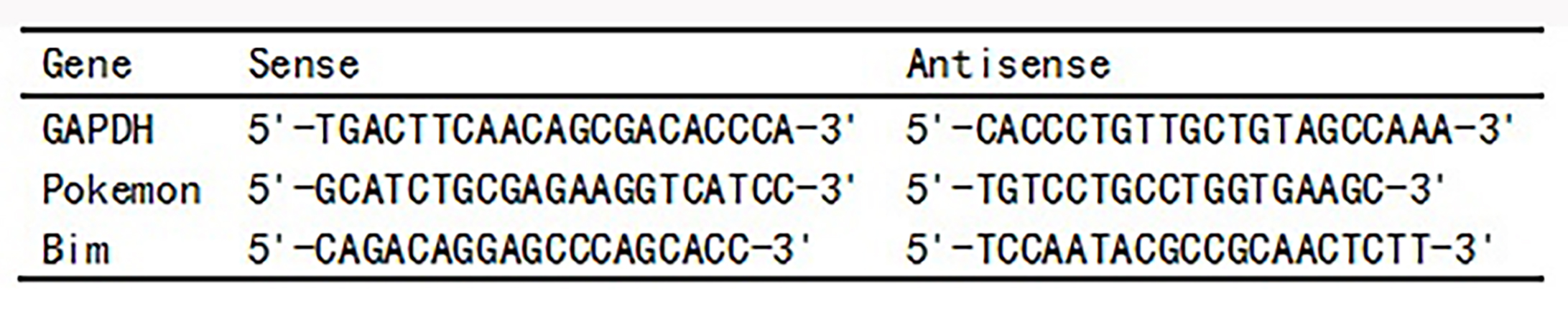

Supplement: Supplementary file 1 — Supplementary Material 1 [file 432_2024_5904_MOESM1_ESM.jpg]

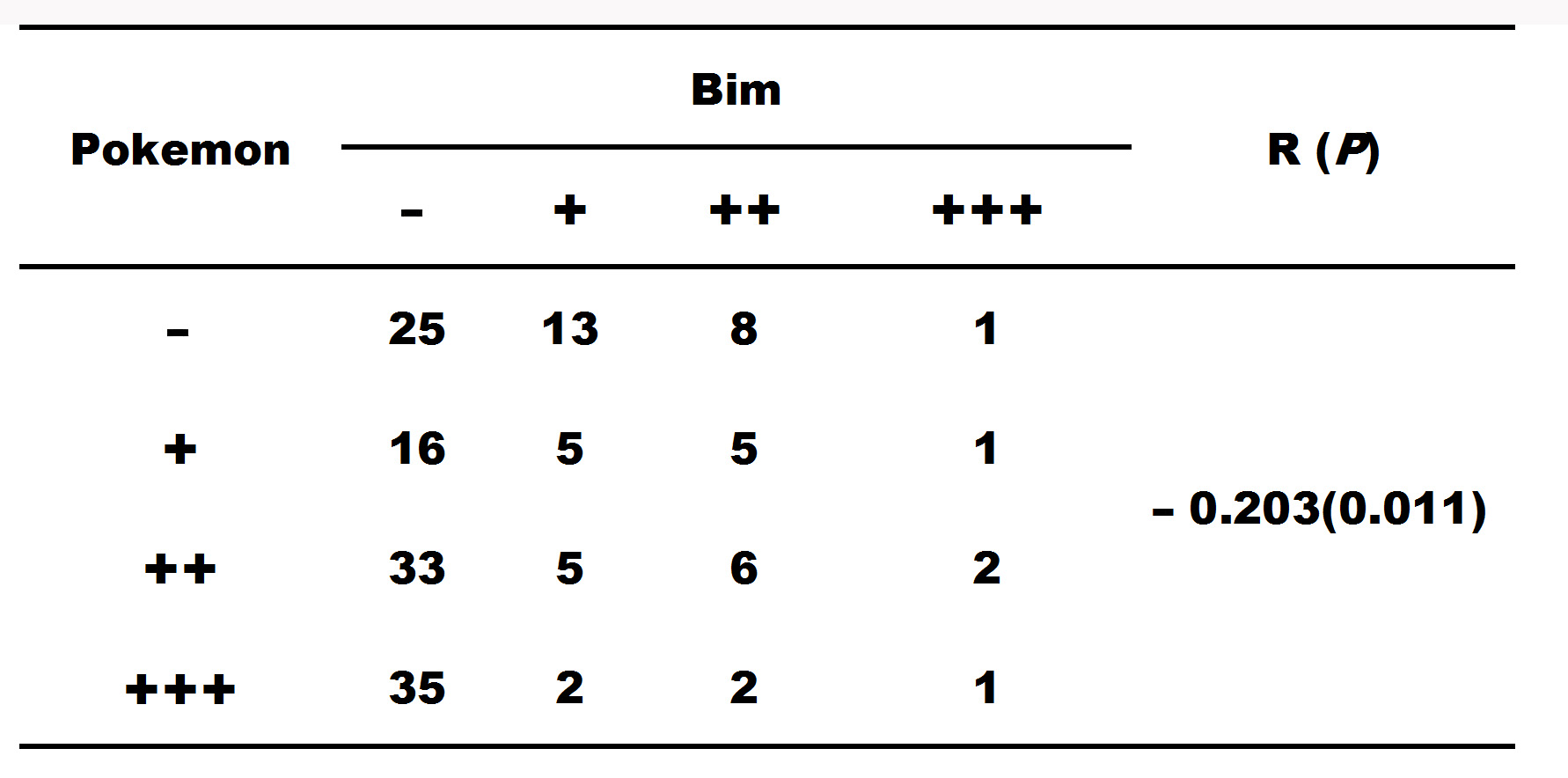

Supplement: Supplementary file 2 — Supplementary Material 2 [file 432_2024_5904_MOESM2_ESM.jpg]
