# Supplementary material for: Pokemon inhibits Bim transcription to promote the proliferation, anti-anoikis, invasion, histological grade, and dukes stage of colorectal neoplasms
Source: J Cancer Res Clin Oncol. 2024 Aug 3;150(8):380. doi: 10.1007/s00432-024-05904-1 (PMC11297103; doi:10.1007/s00432-024-05904-1)
Supplement: Supplementary file 3 — Supplementary Material 3: Table S1. The sequences of primers used for qRT-PCR. Table S2. The pearson correlation analysis of expression between Pokemon and Bim in CRN [file 432_2024_5904_MOESM3_ESM.docx]

**Supplementary Informations**

**TableS1. The sequences of primers used for qRT-PCR**

| Gene | Sense | Antisense |
| --- | --- | --- |
| GAPDH | 5'-TGACTTCAACAGCGACACCCA-3' | 5'-CACCCTGTTGCTGTAGCCAAA-3' |
| Pokemon | 5'-GCATCTGCGAGAAGGTCATCC-3' | 5'-TGTCCTGCCTGGTGAAGC-3' |
| Bim | 5'-CAGACAGGAGCCCAGCACC-3' | 5'-TCCAATACGCCGCAACTCTT-3' |

GAPDH, glyceraldehyde-3-phosphate dehydrogenase.

**TableS2. The pearson correlation analysis of expression between Pokemon and Bim in CRN**

| **Pokemon** | **Bim** | | | | **R(*P*)** |
| --- | --- | --- | --- | --- | --- |
|  | **−** | **+** | **++** | **+++** |  |
| **−** | **25** | **13** | **8** | **1** | **−0.203(0.011)*** |
| **+** | **16** | **5** | **5** | **1** |  |
| **++** | **33** | **5** | **6** | **2** |  |
| **+++** | **35** | **2** | **2** | **1** |  |

*Fisher exact probabilities test.
